# A Re-Interpretation of the Eocene Anuran *Thaumastosaurus* Based on MicroCT Examination of a ‘Mummified’ Specimen

**DOI:** 10.1371/journal.pone.0074874

**Published:** 2013-09-25

**Authors:** Fabien Laloy, Jean-Claude Rage, Susan E. Evans, Renaud Boistel, Nicolas Lenoir, Michel Laurin

**Affiliations:** 1 Muséum national d’Histoire naturelle, Unité mixte de recherche 7207, Centre national de la recherche scientifique/Muséum national d’histoire naturelle/Université Pierre et Marie Curie, Paris, France; 2 Department of Cell and Developmental Biology, University College London, London, United Kingdom; 3 Institut International de Paléoprimatologie et de Paléontologie Humaine, Unité mixte de recherche 7262 Centre national de la recherche scientifique, Université de Poitiers, Poitiers, France; 4 Laboratoire Navier, Unité mixte de recherche 8205, Centre national de la recherche scientifique, École nationale des ponts et chaussées, Institut français des sciences et technologies des transports, de l’aménagement et des réseaux, Marne-la-Vallée, France; College of the Holy Cross, United States of America

## Abstract

What originally appeared to be only an external cast of an anuran ‘mummy’ from the Quercy Phosphorites (southwestern France) was described as *Rana plicata* during the 19th century. Its geographical provenance is only vaguely known; therefore its precise age within the Paleogene was uncertain. The taxon was erected on the basis of the external morphology of the specimen, which includes few diagnostic characters. As a further complication, the name *Rana plicata* was recently shown to be unavailable at the time of the description, and the name *Rana cadurcorum* was proposed as a replacement. In order to see whether internal features were fossilized, the fossil was CT scanned. This showed that a large part of the skeleton is preserved. Unexpectedly, the scans revealed that the skull of the mummy is almost identical to that of *Thaumastosaurus gezei*, another anuran from the late middle or late Eocene of the Quercy Phosphorites. The few observed differences are attributable to intraspecific and ontogenetic variation, and *R*. *cadurcorum* is a junior subjective synonym of *T*. *gezei*. The mummy is therefore probably from the same time interval as *T*. *gezei*. The latter was previously known only by its skull, but the mummy provides important information on the postcranial skeleton. Earlier assessments, based only on the skull, placed *Thaumastosaurus* close to South American hyloid anurans, but a new phylogenetic analysis including postcranial characters reveals ranoid affinities. This study exemplifies the usefulness of modern imaging technologies that allow non-destructive study of previously inaccessible internal anatomical features.

## Introduction

The Quercy Phosphorites are phosphatic clays found in fissures in the limestone plateaux of the Quercy, a region in south-western France. These sediments have produced rich and diverse vertebrate faunas [1] whose ages range from Early Eocene [2] to Early Miocene [3], most localities ranging from late Middle Eocene to Late, but not latest, Oligocene. Fossils are generally represented by disarticulated bones, but very rare external casts (of presumed phosphatic calcium) of amphibians and squamates were recovered. These specimens were generally referred to as ‘mummies’. They were all depicted by Filhol [4]. Unfortunately, these specimens were collected during the 19^th^ century, when fossils from all fissures known at that time were mixed as a single Quercy collection, the so-called ‘old collections’ [5]. Consequently, the geological age of the mummies is unknown.

In 1873, Filhol [6] briefly described, but did not name, a mummified specimen consisting of the anterior part of the body of a frog that lacked the forelimbs (specimen now numbered MNHN [Muséum National d’Histoire Naturelle, Paris] QU 17279). Filhol suggested that a separate forelimb (now numbered MNHN QU 17280) might belong to the ‘same animal’ (he did not specify if ‘same animal’ meant the same taxon or same individual). Subsequently, Filhol [7] erected the name *Rana plicata* for the mummy QU 17279 (he did not allude to the forelimb QU 17280). In 1877, Filhol [4] illustrated the fossils; he referred to both the mummy (QU 17279) and forelimb (QU 17280) as *Rana plicata* and he attributed another external cast (a head and anterior trunk) to this species. Among the mummified specimens, only QU 17279 and QU 17280 (examined using tomography here) are discussed below.

Subsequently, Martín et al. [8] noted that the name *Rana plicata* is a junior homonym of *Rana plicata* Daudin, 1802 [9], which is itself a junior subjective synonym of *Pelodytes punctatus*, an extant European pelodytid frog. Therefore, they proposed *Rana cadurcorum* as a replacement name.

We CT scanned the mummified holotype of *Rana cadurcorum* (QU 17279) and the associated forelimb (QU 17280) and revealed that the skeleton is preserved internally. Examination of the skull of QU 17279 showed it to be almost identical to that of *Thaumastosaurus gezei*, another frog from the Quercy Phosphorites. Thus, the replacement name *Rana cadurcorum* is here considered a junior subjective synonym of *Thaumastosaurus gezei*. The latter was formerly known by only one specimen, a rather complete skull (MNHN QU 17376), which is the holotype of the species. Like QU 17279, the skull of *T. gezei* (QU 17376) was found among fossils of the ‘old collections’ [10]; in other words, its precise geological age is unknown. Aside from *T*. *gezei*, *Thaumastosaurus* includes three species: *T*. *bottii*, *T*. *wardi* and *T*. *sulcatus*. Based on disarticulated bones from the Quercy Phosphorites (*T*. *bottii*; [11]) and from southern England (*T*. *wardi*, *T*. *sulcatus*; [12], [13]), these three species are dated from the Late Eocene (level MP 17). In addition, bones belonging to *Thaumastosaurus* sp. (although formerly referred to *Thaumastosaurus bottii*; [5]) were recorded from the late Middle Eocene (MP 16) to the latest Eocene (MP 19, perhaps MP 20). Therefore, we suspect that the *T. gezei* skull QU 17376 comes from this time interval (MP 16-MP 19 or 20).

The skull preserved in the mummy QU 17279 is more complete than QU 17376. It preserves the snout (including the premaxillae, vomers, anterior parts of nasals), which is lacking in QU 17376. In addition, the maxillae of QU 17279 are entirely preserved, whereas only the mid-portion of one maxilla remains in QU 17376. Moreover, some bones of the hyobranchial apparatus and a large part of the postcranial skeleton are preserved in the mummy QU 17279. Previous studies have suggested that *Thaumastosaurus* is more or less closely related to South American hyloid frogs [10], [14], but these phylogenetic hypotheses were based only on available skull characters. The new cranial and postcranial data revealed by QU 17279 permit a comprehensive reassessment of the phylogenetic relationships of *Thaumastosaurus*.

The separate forelimb QU 17280 is also described below, but it is not possible to state definitely whether or not it belongs to *T*. *gezei*.

## Materials and Methods

**Figure 1:**
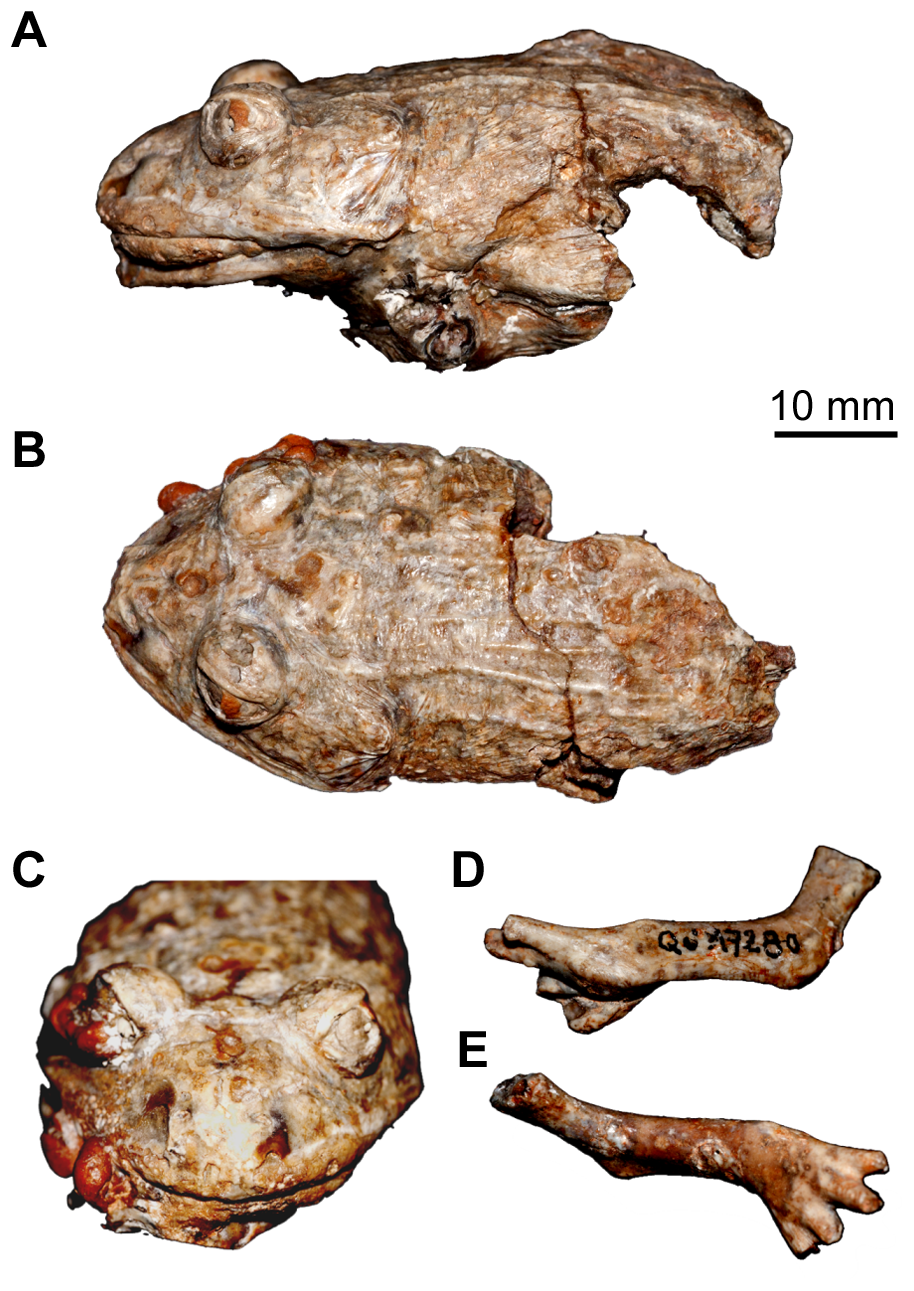
External casts of the mummy MNHN QU 17279, and the separate forelimb MNHN QU 17280. The mummy QU 17279 is shown in left lateral (**A**), dorsal (**B**), and anterior (**C**) views. The forelimb QU 17280 is seen in left lateral (**D**), and dorsal (**E**) views.

The mummy (MNHN QU 17279) (Figure 1A–C) and the forelimb (MNHN QU 17280) (Figure 1D, E), both currently displayed in the palaeontology gallery of the Natural History Museum (Paris), were scanned at the Laboratoire Navier (Marne-la-Vallée, France) as part of a Master’s project [15]. A Ultratom RXsolutions microtomograph was used, with an acceleration tension of 120 kV and an intensity of 180 µA. 1440 radiographs were acquired with an exposure time of 0.25 s and an averaging of 16. The resolution was 25 µm. Two scans with a Z-translation were done in order to acquire the whole specimen.

Image segmentation was performed using Avizo v.6.3 (Visualization Sciences Group, Inc.) on the CeMIM (Centre de Microscopie de fluorescence et d’Imagerie numérique) computers of the MNHN. For each bone, the pixels forming the region of interest, identified by contrast on the grey-scale images, were manually selected on slices with the contouring tool; the process was repeated every 5 or 10 slices, sometimes less, depending on how much the structure changed from slice to slice, and interpolation was used between the slices to create the volume of interest. The isosurface function was then applied to build a smooth 3 D surface from a subset of selected voxels of each bone.

**Figure 2:**
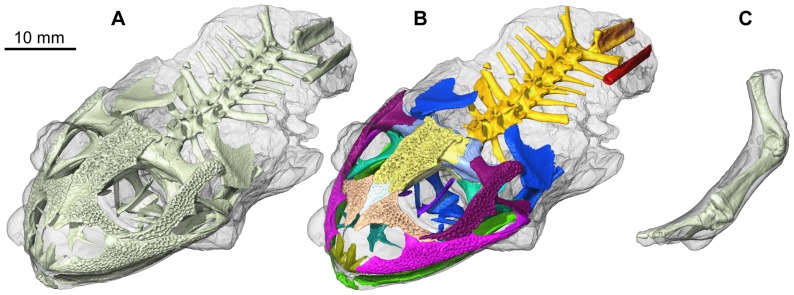
3D renderings of the whole skeleton of the mummy QU 17279. The skeleton is visible through the external cast, shown in transparency. These and all 3D models hereafter presented were reconstructed using Avizo v.6.3 (Visualization Sciences Group, Inc.). **A**, skeleton, uniformly coloured. **B**, skeleton, with the different regions and bones displayed in different colors. **C**, separate left forelimb QU 17280.

The tomogram revealed that a few structures composed of soft tissues (e.g. brain, spinal cord) are preserved, although they are either too degraded, or similar to other materials in terms of density to be successfully segmented and thus to be described here. These elements are not included in our description, which focuses on the skeleton. The latter is well preserved, apart from the pelvic region and the limbs, because QU 17279 is incomplete (Figure 2A–C). Many elements appear in situ and are articulated, but some of them have moved slightly relative to one another.

The anatomical terminology used hereafter is based on Roček [16].

No permits were required for the described study, which complied with all relevant regulations.

## Results and Discussion

### Systematic Palaeontology

Anura Fischer von Waldheim, 1813 [17]

Ranoides Frost et al., 2006 [18]

Natatunara Frost et al., 2006 [18]

*Thaumastosaurus gezei* Rage and Roček, 2007 [10]

*Rana plicata* Filhol, 1876 [7]

non *Rana plicata* Daudin, 1802 [9]

*Rana cadurcorum* Martín et al., 2012 [8]

Examined material: MNHN QU 17279, mummy preserving most of the body except for the appendages (Figure 1A–C); MNHN QU 17280, forelimb (Figure 1D, E); MNHN QU 17376, holotype of *Thaumastosaurus gezei,* consisting of an incomplete skull.

Locality and age: old collections of the Quercy Phosphorites (precise localities unknown), probable late middle and/or late Eocene (see below).

### Description

#### Skull

Features of the mummy QU 17279 previously recorded from the original *T. gezei* skull QU 17376

**Figure 3:**
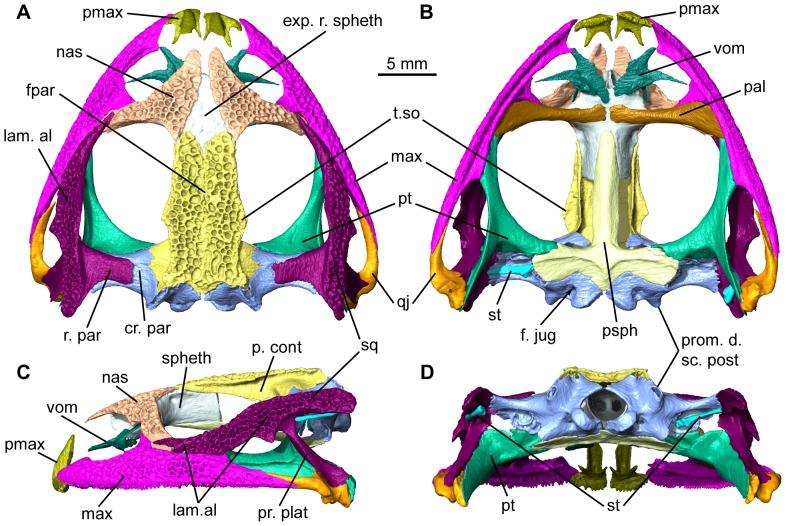
Complete articulated skull of the mummy QU 17279. Shown in dorsal (**A**), ventral (**B**), left lateral (**C**), and posterior (**D**) views. Each bone or paired bone is identified with a single color for easy delimitation. Anatomical abbreviations: cr. par, *crista parotica*; exp. r. spheth, exposed rhomboid area of sphenethmoid; f. jug, *foramen jugulare*; fpar, frontoparietals; lam. al, *lamella alaris*; max, maxilla; nas, nasal; p. cont, *pars contacta*; pal, palatine; pmax, premaxilla; pr. plat, *processus posterolateralis*; prom. d. sc. post, *prominentia ducti semicircularis posterioris*; psph, parasphenoid; pt, pterygoid; qj, quadratojugal; r. par, *ramus paroticus*; spheth, sphenethmoid; sq, squamosal; st, stapes; t. so, *tectum supraorbitale*; vom, vomer.

Complete and articulated, the skull of QU 17279 is almost as long as wide (Figure 3A–D). A dermal sculpture covers the frontoparietals, squamosals, nasals and maxillae, consisting of subcircular to suboval pits limited by low ridges of more or less constant thickness. A second diagnostic character is the *lamella alaris* of the squamosal, which has an anteriorly elongated projection; the latter rests on the dorsal margin of the maxilla, thus separating this bone from the orbit (Figure 3A, C). The prootic and palatine foramina open in the posteromedial wall of the orbit and a shallow groove for the *vena jugularis interna* runs posterolaterally to the two foramina. The *ramus paroticus* of the squamosal consists of a broad lamina solidly sutured to the *crista parotica* of the otic capsule, on its dorsal and anterodorsal surfaces. The posterolateral process of the squamosal, extending from the *ramus paroticus* to the *crista parotica* of the otic capsule, forms a lamina that makes up the lateral part of the posterior orbital wall. The internal ramus of the pterygoid is also united with both the otic capsule and the posterolateral process of the parasphenoid by a suture showing deep and narrow interdigitations. This complex configuration was noticeable in section during segmentation, but is extremely hard to model due to the slenderness of the structure. There is no contact between the squamosal and the frontoparietal. The latter lacks a visible sagittal suture, unless it remained unnoticed on the tomograms; however, the sculpture is weaker along the midline than elsewhere on the dorsal surface. On either side, the lateral margin of the frontoparietal extends as a *tectum supraorbitale*. Ventrally, the *pars contacta* is well developed (Figure 3C). A small portion of the dorsal surface of the sphenethmoid is exposed in a rhomboid space between the frontoparietal and the nasal (Figure 3A). The anteriormost part of the sphenethmoid is clearly damaged, but it probably did not extend anteriorly much beyond its preserved part. On the ventral face of the skull, the palatines are present and are not separated from one another by the anterior tip of the parasphenoid; however, the palatines do not make contact in the midline (Figure 3B). The posterior area of the skull closely resembles that of QU 17376. The condyloid fossae, in which the jugular foramina open, are partially hidden by the occipital condyles. The posterior openings of the canals for the occipital arteries open just below the sculptured frontoparietal table and the *prominentia ducti semicircularis posterioris* on each side is compressed mediolaterally and projects strongly posteriorly (Figure 3D).

#### Differences between the skull of QU 17279 and QU 17376

In spite of the many common characters shared by QU 17279 and QU 17376, there are a few notable differences. Although very elongate, the *lamella alaris* is shorter in QU 17279 than in QU 17376 and it does not separate the maxilla from the nasal [10]. The nasals, which are fused in the midline in QU 17376, are markedly separated in the midline in the scanned specimen (Figure 3A). The rhomboid area between the frontoparietal and the nasal is barely sculptured in QU 17279, whereas in QU 17376 the sculpture is clearly apparent although less marked than elsewhere. The palatine in QU 17279 does not reach the midline, nor does it contact the anterior tip of the parasphenoid (Figure 3B), contrary to QU 17376. On the posterolateral wall of the orbit, the groove for the *vena jugularis interna* is wider and not as well defined in QU 17279 as in QU 17376, and two unnamed foramina found in QU 17376 are not observed in QU 17279 ([10]: Fig. 7A). The condyloid fossae are larger in QU 17279 and not as well delimited as in QU 17376. QU 17279 also differs from QU 17376 in lacking a horizontal groove containing a minute foramen above the condyloid fossa QU 17376. However, based on comparisons with extant frogs [19], [20], [21], most or all of these differences can be attributed to ontogeny: we interpret QU 17279 as a post-metamorphic individual, yet younger than the presumably mature adult specimen represented by QU 17376. This conclusion is supported by the patent notochordal canal in presacral VIII of QU 17279.

#### New information on the skull of *Thaumastosaurus*

**Figure 4:**
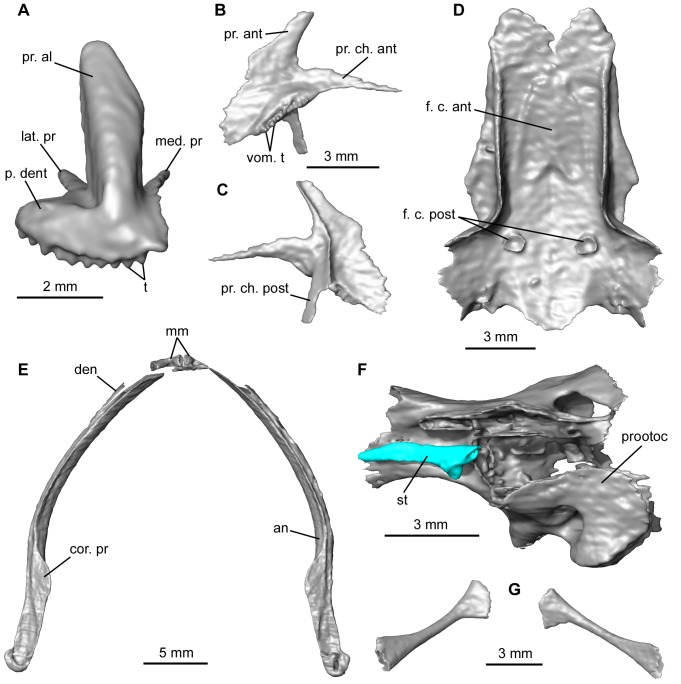
Some of the new elements provided by the skull of the mummy QU 17279. **A**, right premaxilla in anterior view. **B–C**, left vomer in ventral and dorsal views. **D**, frontoparietals in ventral view. **E**, lower jaw in dorsal view. **F**, right prooticooccipital and stapes in ventral view. **G**, thyrohyal ossifications of the posteromedial processes of the hyoid (in anatomical position). Anatomical abbreviations: an, angular; cor. pr, coronoid process; den, dentary; f. c. ant, *facies cerebralis anterior*; f. c. post, *facies cerebrales posteriores*; mm, mentomeckelian bones; med. pr, medial process; lat. pr, lateral process; p. dent, *pars dentalis*; pr. al, *processus alaris*; pr. ant, *processus anterior*; pr. ch. ant, *processus choanalis anterior*; pr. ch. post, *processus choanalis posterior*; prootoc, prooticooccipital; st, stapes; t, teeth; vom. t, vomerine teeth.

Several cranial features found in QU 17279 were not preserved in QU 17376, most notably those of the snout region. The premaxillae are slightly disarticulated, and each bears pleurodont teeth all along its *pars dentalis*; the right premaxilla, on which they are more clearly seen, had twelve tooth loci, ten of which are filled (Figure 3B, Figure 4A). Located medially, the *processus alaris* is tall and straight, inclined posteriorly, concave lingually at its base and then flattening towards its dorsal narrower extremity. The short *pars dentalis* projects posterolaterally. Lingually, two processes originate from the *lamina horizontalis*. The medial process is slender and directed posteriorly, whereas the lateral one, longer and thicker, is directed posterolaterally. In anatomical position, the posterolateral margin of the *pars dentalis* was overlapped by the anterior tip of the maxilla. The latter, complete in the scanned specimen, is posteriorly in contact with the quadratojugal (Figure 3A–C). A *processus zygomatico-maxillaris* does exist, as well as a developed *processus frontalis*. Teeth are present, although not easily individually distinguishable and showing few details, preventing us from determining whether they were uni- or bicuspid, and whether or not they were pedicellate. The right maxilla, where they can be more easily counted, bears at least 62 teeth.

The vomer is preserved and is articulated with the ventral face of the sphenethmoid (Figure 3B). It is paired, separated from the midline, moderately extended, and bears an oblique series of very small teeth on its ventral face (at least seven on the left vomer, where they are more clearly seen) (Figure 4B, C). The anterior process forms a small elongated, tapering lamina that extends anterolaterally toward the *lamina horizontalis* of the maxilla and contacts it; the anterior *processus choanalis* is markedly elongate and is directed laterally, but it did not reach the *lamina horizontalis* of the maxilla; the posterior *processus choanalis* is a flat lamina that is directed posterodorsally. Both anterior and posterior processes bear a crest on their dorsal face. Each vomer is posteromedially extended by another process consisting of a flat lamina whose posterior border is rounded and approaches the palatine without touching it.

On the ventral face of the frontoparietal, the *incrassatio frontoparietalis* is comprised of an anterior, lanceolate unpaired *facies cerebralis anterior* and two small and circular *facies cerebrales posteriores* that are somewhat distant from the anterior facies (Figure 4D). The incrassations are very slight thickenings of the ventral face of the frontoparietal that insert into fenestrae of the endocranial roof [22]; therefore, they mirror the topography of the dorsal endocranium and may be phylogenetically informative. Unfortunately, their morphology and distribution within anurans are still poorly known.

All constituents of the lower jaw are preserved: the paired angular and dentary, and mentomeckelian bones, the latter being disarticulated relative to the rest (Figure 4E). As is typical in anurans, the meckelian groove runs on the lateral face of the angular, except in the posterior portion of the bone where the groove passes on the dorsal face. The dentary lacks odontoids. The posterior extremity of the angular rises abruptly. A coronoid process is present on the angular; it is inclined mesially at ca 45°. The mentomeckelian bones are not conjoined, i.e. the symphysis is not fused. A strip of unidentified matter connects the posterior face of the mentomeckelian on both sides, but it is probably a remnant of sedimentary matrix that could not be distinguished from the bony structures during segmentation.

The otic capsules are damaged and ventrally poorly ossified, but the stapes is preserved on both sides (Figure 3B, D). The right stapes is almost in functional position, ventral to the *crista parotica* of the capsule (Figure 4F). The stapes consists of a small thickened footplate (*pars interna plectri*) that was ossified; it is prolonged dorsolaterally by a long rod (*pars media plectri*), which is slightly curved anteriorly. The cartilaginous distal part (*pars externa plectri*) of the stapes and *annulus tympanicus* [23] are not preserved, assuming they existed, or may have not been visible during segmentation due to problems of contrast with surrounding materials having similar densities. The medial face of the footplate of the stapes appears to be transversely depressed; in addition, its rim is shallowly notched posteroventrally. The *foramen ovale* is larger than the footplate, which suggests that a cartilaginous operculum was present; this is supported by the posteroventral indentation of the footplate rim, the anterior border of the operculum generally being inserted into a stapedial notch like this.

#### Hyobranchial skeleton

No cartilaginous part of the hyoid apparatus was found. However, the ossified parts ( =  thyrohyal bones) of the posteromedial processes are present and, although they are isolated, seem to be directed posterolaterally (Figure 4G). They widen both proximally and distally. There is no ossified parahyoid.

#### Postcranial skeleton

**Figure 5:**
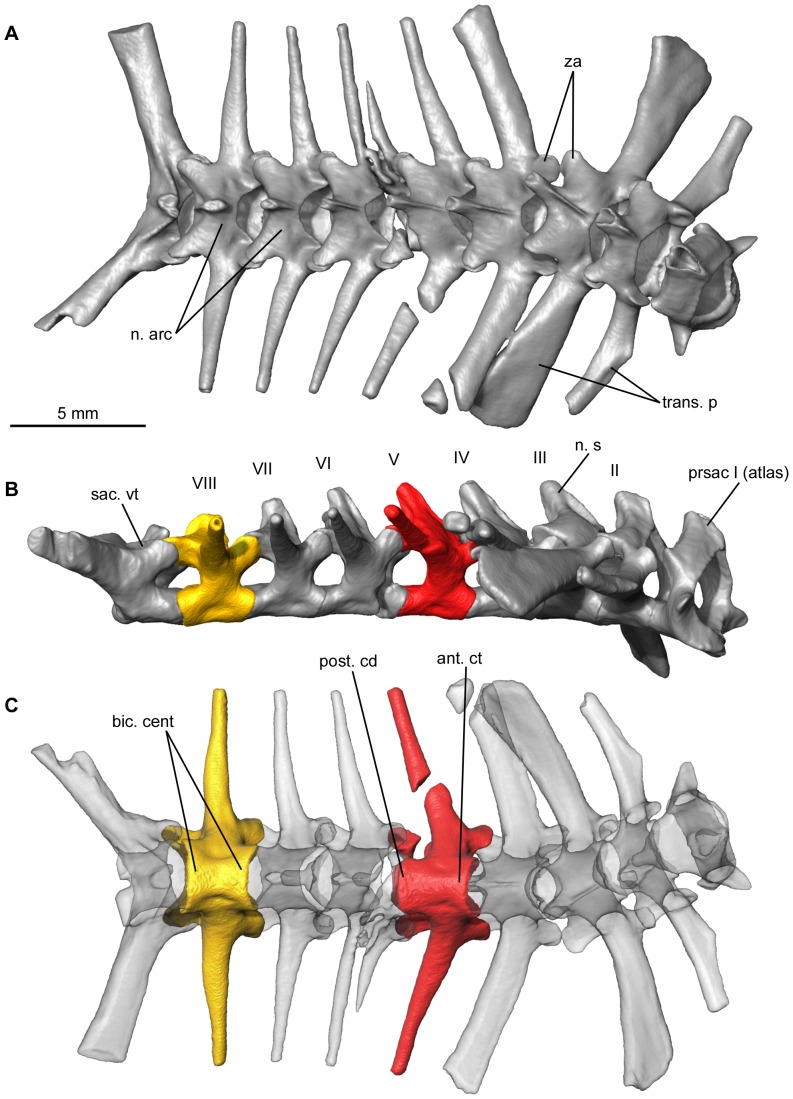
Articulated diplasiocoelous vertebral column of the mummy QU 17279, with the exception of the urostyle. **A**, dorsal view. **B**, right lateral view. **C**, ventral view, with transparency. **B** and **C** show a procoelous vertebra (in red) and the amphicoelous last presacral (in yellow). Anatomical abbreviations: ant. ct, anterior cotyle; bic. cent, biconcave centrum; n. arc, neural arches; n. s, neural spine; post. cd, posterior condyle; prsac, presacral; sac. vt, sacral vertebra; trans. p, transverse process; za, zygapophyses.

**Figure 6:**
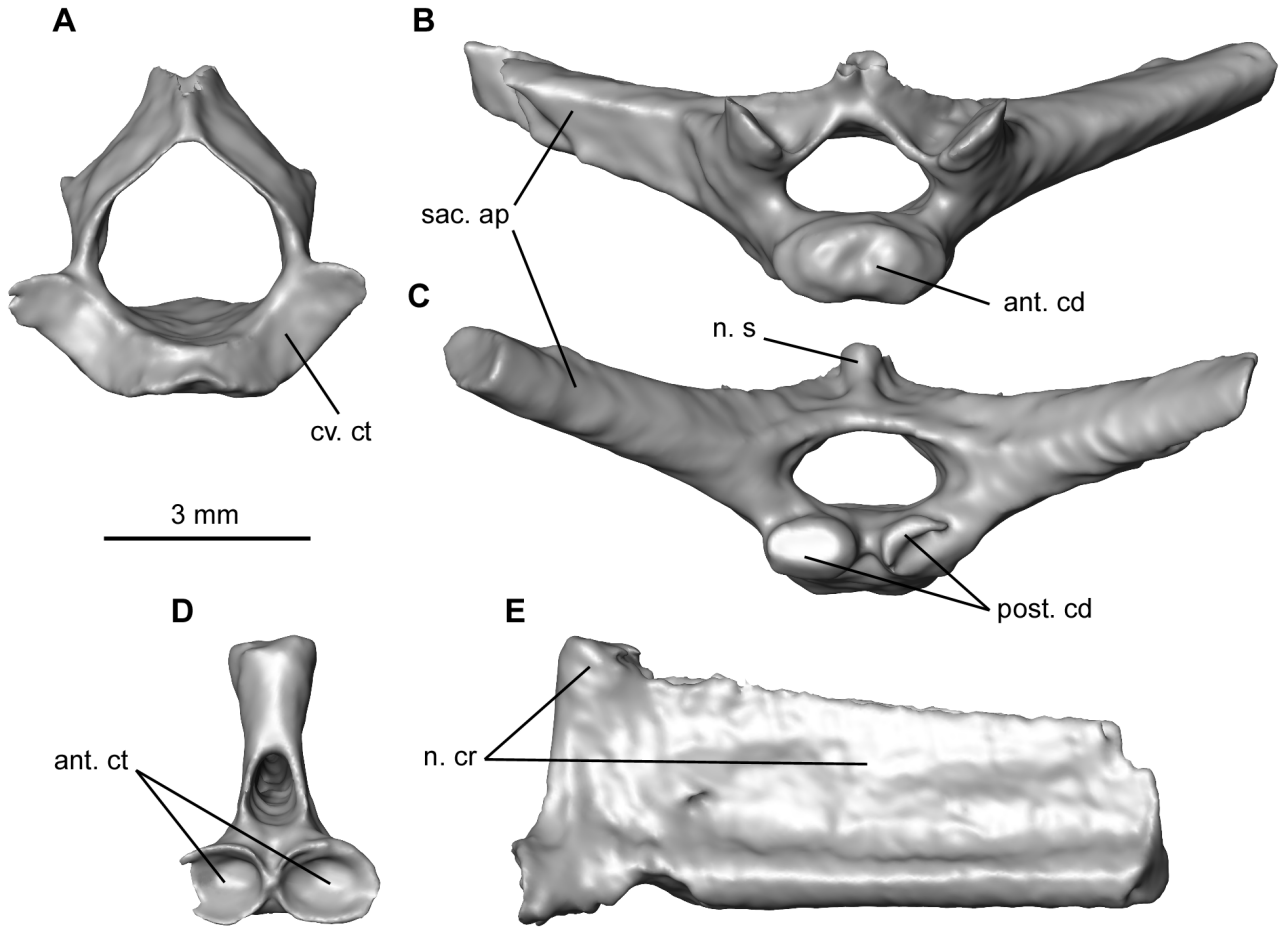
Some elements of the vertebral column of the mummy QU 17279. **A**, atlas in anterior view. **B–C**, sacral vertebra in anterior and posterior views. **D–E**, urostyle lacking the posterior part, in left lateral and anterior views. Anatomical abbreviations, as in Figure 5, and: ant. cd, anterior condyle; cv. ct, cervical cotyle; n. cr, neural crest; sac. ap, sacral apophysis.

The vertebral column possesses eight presacral vertebrae, one sacral vertebra and a urostyle (Figure 5A–C, and Figure 6D, E). The atlas articulates with the skull by means of two elongate, apparently confluent oval cervical cotyles (type III of Lynch [24]) (Figure 6A). The posterior face of the atlas centrum is convex. The base of the incomplete neural spine is thick. Its postzygapophyses are strongly inclined. The massive post-atlantal vertebrae have a dorsoventrally depressed centrum and are all procoelous, except the last presacral (i.e., vertebra VIII), which is biconcave (Figure 5C) and retains a notochordal canal. The vertebral column is therefore diplasiocoelous following the terminology of Nicholls [25] and Noble [26]. The centra of presacrals III–VIII are constricted at mid-length and are therefore more or less hourglass-shaped. The neural canal is broad, particularly on the anterior vertebrae. The neural arches are of the non-imbricate type and leave a dorsal space between adjacent vertebrae, but they are not strongly shortened (Figure 5A). The neural spines are tall and well developed, but do not extend further than the posterior margin of the postzygapophyses (Figure 5B). The dorsal and posterior extension of the neural spines is weaker in the last presacrals, and there is even a slight anterodorsal tilt of the neural spine in presacral VIII. The zygapophyses have flat articular surfaces. Presacrals II–VII possess long transverse processes that, apart from those of presacral II, extend further laterally than the tips of the sacral apophyses. Those of the third vertebra are oriented ventrally and expanded distally, whereas presacral IV has transverse processes that are directed posteriorly and do not widen distally. On presacrals V–VIII, the transverse processes are much thinner and narrow distally. They are perpendicular to the axial axis on presacrals VI-VIII. No vertebra bears ribs.

The sacral vertebra has two distinct posterior condyles that articulate with the urostyle and a larger anterior condyle articulating with the posterior cotyle of the last presacral. This arrangement is typical of the diplasiocoelous configuration (Figure 6B, C). The sacral apophyses are approximately cylindrical, slightly depressed, project slightly posteriorly, and do not widen distally. An anterodorsally inclined neural spine is present. On either side, a well-developed ridge joins the anterior part of the neural spine to the base of the sacral apophysis.

The posterior part of the urostyle is missing and what remains could represent less than one half of the complete element (Figure 6D, E). It is not fused to the sacral vertebra, to which it was articulated through two oval cotyles whose rims merge medially. No anterior transverse processes are present. The neural arch bears a tall neural crest. The anteriormost part of this crest thickens dorsally, but the crest becomes thinner and shallower posteriorly and a sagittal groove runs on its dorsal margin.

**Figure 7:**
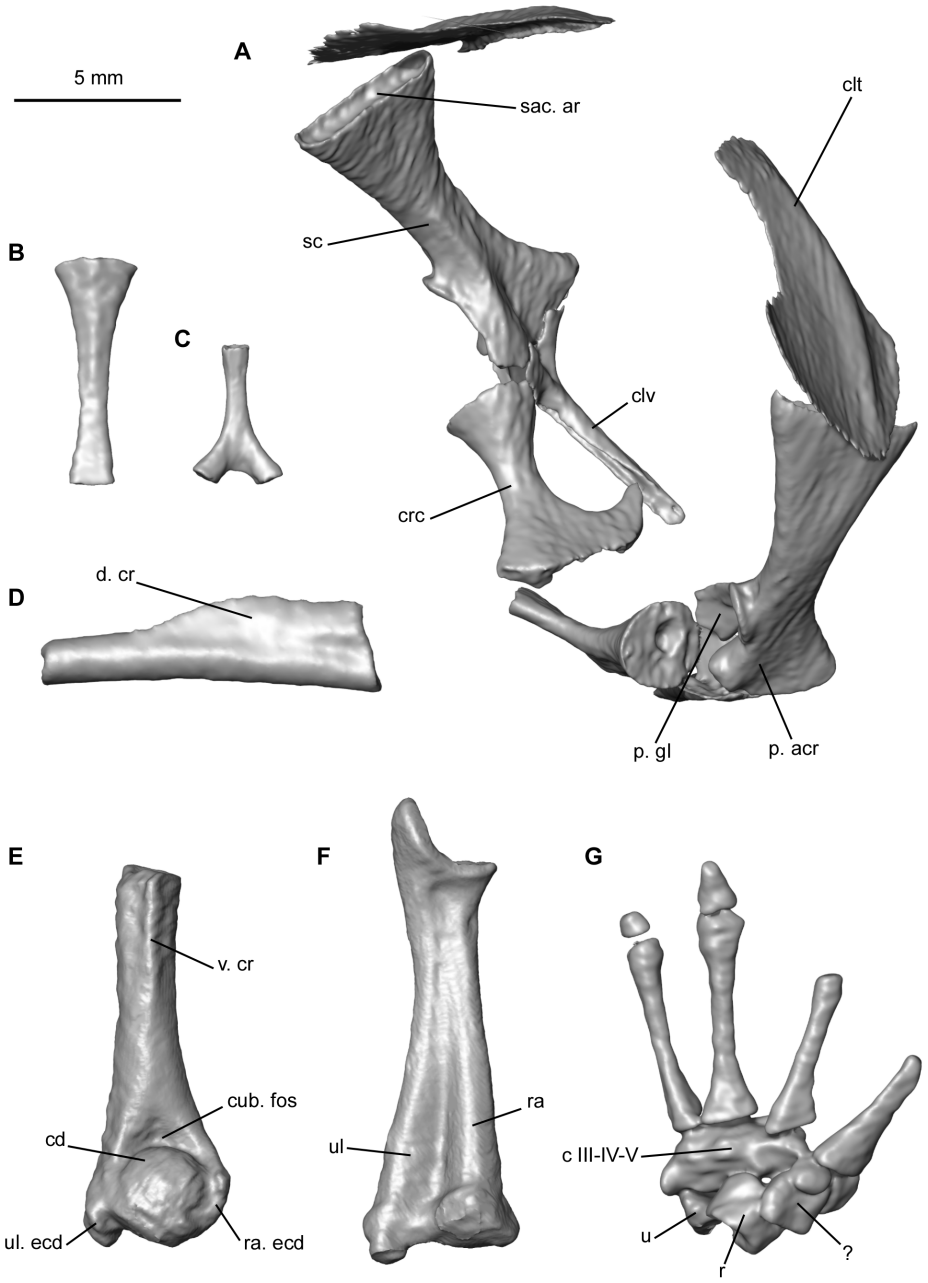
Appendicular skeleton of the mummy QU 17279 and bones of the forelimb QU 17280. The pectoral girdle is illustrated in posterolateral view (**A**), sternum in ventral view (**B**), and omosternum in dorsal view (**C**). The pelvic girdle is represented by the anterior portion of the shaft of the left ilium (**D**). QU 17280 comprises the distal portion of the left humerus in ventral view (**E**), the left radioulna in posterior view (**F**), and the left manus in dorsal view (**G**). Anatomical abbreviations: c, carpal; cd, condyle; clt, cleithrum; clv, clavicle, crc, coracoid, cub. fos, cubital fossa; d. cr, dorsal crest; p. acr, *pars acromialis*; p. gl, *pars glenoidalis*; r, radiale; ra, radius; ra. ecd, radial epicondyle; sc, scapula; ssc. ar, suprascapula articulation; u, ulnare; ul, ulna; ul. ecd, ulnar epicondyle; v. cr, ventral crest.

The cartilaginous components of the pectoral girdle were not visible during segmentation. Both halves of the girdle have moved slightly relative to one another (Figure 7A). The scapula is dorsoventrally tall and it broadens dorsally. Its anterior border is concave, whereas the posterior border is straighter. Ventrally, the *pars acromialis* and the *pars glenoidalis* are separated by a moderately wide notch. The former is more developed than the latter, lacks a posterior protuberance and has rounded anterior and ventral edges. A groove on the dorsal margin of the scapula forms the articulation for the suprascapula. The clavicle is straight and is directed posterolaterally. Its dorsolateral extremity broadens and bifurcates; it contacts both the *pars acromialis* and *pars glenoidalis*. The coracoid is almost as long as the clavicle, with which it articulates dorsolaterally. The coracoid is not in contact with any of the scapular apophyses, but this probably results from post-mortem displacement. Its dorsolateral end is thickened and circular in cross-section; the ventromedial extremity is flat and expanded, forming a hook anteriorly that approaches but does not contact, the clavicle. The coracoids are almost in contact with each other ventrally through these extremities. Overall, this is the typical morphology of a firmisternal pectoral girdle type sensu Cope [27] and Boulenger [28]. Dorsally, the ossified cleithrum forms a broad plate, with an anterior portion that is slightly expanded medially.

Two disarticulated midline elements are also preserved. The posterior element, the sternum, consists of an elongate ossified rod that expands slightly anteriorly (Figure 7B). The anterior element, the omosternum, is also ossified and exhibits a markedly forked posterior extremity (Figure 7C). The presence of the latter bone also denotes the firmisternal condition of the girdle.

The proximal end of the humerus is preserved on both sides in the mummy QU 17279, but it is badly damaged. The proximal part of the right humerus shows the beginning of a moderately developed ventral crest.

The separate left forelimb QU 17280 consists of a humerus lacking its proximal portion, the radioulna, and the manus, for which phalanges are missing (Figure 7E–G). It is difficult to determine whether the diaphysis of the humerus was straight or slightly curved as the bone is not complete (Figure 7E). It possesses a large hemispherical condyle, proximal to which is a marked, triangular proximodistally elongate cubital fossa. Of the two epicondyles, the radial epicondyle is smaller than the ulnar one, and neither one bears a crest. The condyle is not exactly in line with the axis of the diaphysis, but it is slightly shifted laterally. The distal limit of the ventral crest lies well proximal to the condyle. The radioulna is moderately long and has a thickened distal extremity (Figure 7F). The ulnar border is slightly curved, whereas the radial border is almost straight. In dorsal view, on the distal portion of the bone, the ulna is shifted laterally, relative to the radius. In the carpus, the bones are small and closely packed, hardly distinguishable from the surrounding tissues and matrix on the tomograms, making them very difficult to segment out fully. A proximal row is formed by the ulnare and radiale; anteromedial to them, is an element that may be a centrale. Carpals III, IV and V appear to be fused but this may be due to lack of resolution during segmentation. The manus has four digits, for which the metacarpals are almost completely preserved (Figure 7G) but the phalanges were lost. There is no trace of prepollex elements.

The only element from the pelvic girdle still present is an anterior portion of the shaft of the left ilium, which is slightly convex dorsally (Figure 7D). Its anterior extremity is positioned under the tip of the sacral apophysis, with which it articulated. Posterior to the articular area, a well-developed, rather tall dorsal crest is present on the shaft; it is tilted medially.

### Taxonomic Status

The mummified specimen QU 17279 is the holotype of *Rana cadurcorum* [8], but its skull is almost identical to that of QU 17376, the holotype of *Thaumastosaurus gezei*. Both differ from *T*. *bottii* [11] in having a long anterior extension of the squamosal that separates the maxilla from the orbit. This character cannot be confirmed directly in *T*. *wardi* [12] because the available bones of this species are disarticulated and incomplete. However, the morphology of the known, but incomplete, squamosal, the *lamella alaris* of which is slender, suggests that an anterior extension was lacking. The state of this character is unknown in *T*. *sulcatus* [13]. QU 17279 further differs from *T*. *wardi* in the convex medial face of the *lamella horizontalis* of the maxilla (flat in *T*. *wardi*) and in the almost straight ridge that forms the base of the *processus paroticus* (markedly curved in *T*. *wardi*). QU 17279 also differs from *T*. *sulcatus* in the pattern of sculpture on the dermal skull bones: approximately circular pits clearly delimited by marked ridges in QU 17279, but most pits are replaced by elongate grooves and ridges in *T*. *sulcatus*. QU 17279 thus differs in individual details from *T*. *bottii*, *T*. *wardi* and *T*. *sulcatus*, and in details not readily attributable to ontogenetic stage. However, QU 17279 does not differ significantly from QU 17376, the holotype of *T*. *gezei*, and it is therefore assigned to *Thaumastosaurus gezei*. The forelimb QU 17280 may also belong to this taxon, but this cannot be confirmed without further associated material.

A consequence of the attribution of QU 17279 to *T*. *gezei* is that *Rana cadurcorum* Martín et al., 2012, is here considered a junior synonym of *Thaumastosaurus gezei* Rage and Roček, 2007. *Rana plicata* Filhol, 1876, as mentioned above, is a junior homonym of *Rana plicata* Daudin, 1802 [9].

### Phylogenetic Relationships

**Figure 8:**
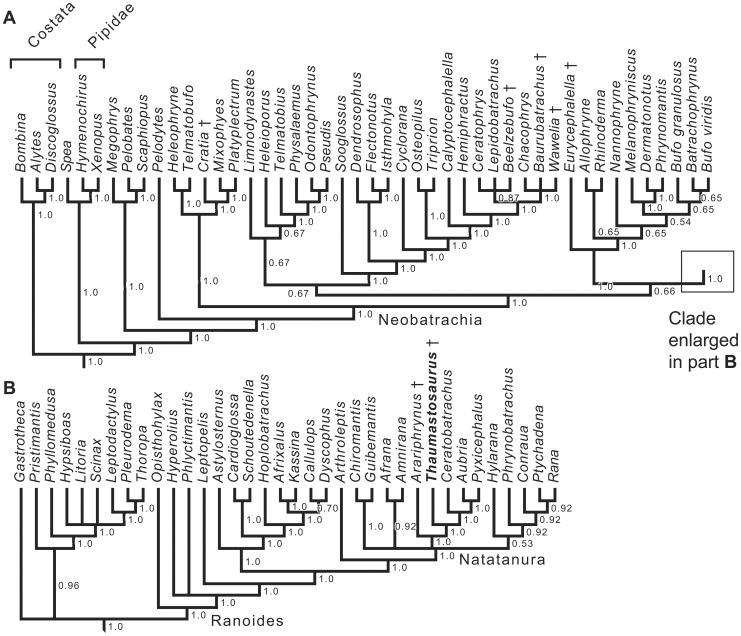
Majority-rule consensus of the 136 211 most parsimonious (MP) trees (709 steps). More trees must exist but the heuristic search had to be interrupted because of memory limitations. The consensus tree is split into two parts (**A** and **B**) for reading convenience. Costata [18] is equivalent to the classical Discoglossidae. Extinct taxa are identified by the symbol †. Numbers at nodes indicate relative clade frequency, rounded off at the second decimal, among these MP trees. These trees were found by PAUP and TNT, their length was compared in Mesquite [36], in which the consensus tree was also computed. See Materials and Methods for more information.

In order to investigate further the relationships of *Thaumastosaurus*, phylogenetic analyses were performed, based on an existing morphological character matrix [29] whose character list was modified from Fabrezi [30] and Evans et al. [14]. Several changes were made in the dataset: 40 taxa from Evans et al. [14], including *Thaumastosaurus*, were added into the matrix, and genera were used instead of species. The character ‘dorsal exposure of sphenethmoid’ was deleted as we regard it as ambiguously defined; consequently, our matrix includes 74 characters. The character ‘expansion of sacral diapophyses’ was reworded, with more details given for the definition of its three states. The 74 characters were equally weighted, and 13 of them, which form morphoclines, were ordered, as this is optimal in such cases [31]. Multiple states in a given cell were considered to represent polymorphism. As we compared fully developed adults, we relied on the holotype of *Thaumastosaurus gezei* (QU 17376) for character 1 (‘nasals medial contact’) in the main analysis, because we interpret the condition in QU 17279 (nasals separated) as reflecting immaturity. However, we repeated the analysis with this character scored from QU 17376 to determine if this influenced the topology using the data matrix S1 (described below), for which we modified a single cell (character 1 in *T. gezei*). The parsimony analyses were performed with PAUP v.4.01b [32], using the heuristic search mode, as well as with TNT [33], using all its new technologies, such as the parsimony ratchet [34], sectorial searches, tree-fusing, and tree-drifting [35]. We then compared the length of trees obtained in TNT and PAUP in Mesquite [36] to ensure that they were of the same length (differences in the treatment of multi-state taxa can create spurious length differences between phylogenetic programs), and used the trees from PAUP as starting point for further searches in TNT. Thus, we are reasonably certain that we found some of the shortest trees, although not all of them, because of memory limitations (typically, we restricted our searches to 100 000–150 000 equally parsimonious trees). Because of the computing time involved (about a week, for the four matrices), it was not possible to conduct bootstrap or decay index searches, which would have been several times more time-consuming to assess with reasonable accuracy. The extant *Bombina*, *Alytes* and *Discoglossus* were used as outgroups. Due to uncertainty related to the link between *Thaumastosaurus* and the separate forelimb QU 17280, as well as an issue of definition for character 52 (‘sternum expansion of the coracoid’), with possibly two non-homologous states included in state 1 (‘more than one half’), four distinct analyses were performed to investigate how such factors may have affected the topology of the resulting trees: the first analysis includes all characters (data matrix S1), the second has the characters of the forelimb QU 17280 coded as missing for *Thaumastosaurus* (Data Matrix S2), the third is without character 52 (Data Matrix S3), and the last one combines both modifications (Data Matrix S4). See character list S1 for character information. The first analysis yielded 136 211 equally parsimonious trees of 709 steps, for which the majority rule consensus tree is shown (CI = 0,1547, HI = 0,8537, RI = 0,5555, RCI = 0,0859) (Figure 8). Character transformations are discussed below in a delayed transformation optimization (DELTRAN) context.

In the strict (and hence, also in the majority-rule) consensus, *Thaumastosaurus* appears within Ranoides (sensu Frost et al. [18]), which is globally recovered in our tree, though the internal topology is significantly different from the one obtained by these authors. Three major characters support this position: the biconcave last presacral vertebra, the cylindrical sacral diapophyses, and the ossified, proximally forked omosternum.

The biconcave eighth vertebra is evidence for the diplasiocoelous condition of the vertebral column. This configuration was defined by Nicholls [25] as follows: the first seven presacrals are procoelous, the eighth vertebra is amphicoelous, and the sacral vertebra centrum is biconvex, bearing two condyles posteriorly and one anteriorly. Most ranoids are reported as diplasiocoelous [18]. The presence of an ossified forked omosternum suggests firmisterny of the pectoral girdle. Traditionally, this type of girdle is recognized by the fusion of the epicoracoid cartilages to each other medially, and the absence of epicoracoid horns [37]. The presence of this condition in QU 17279 is further corroborated by the straight clavicles that match the coracoids in length, and by the close approximation of the clavicle and coracoid ventrally. An ossified omosternum has already been proposed as an apomorphy of Ranoides [18], and the firmisternal girdle type has long been associated with ranoids [38]. Cylindrical sacral apophyses are recognized as a predominant feature in ranoids [39], [40].

Within Ranoides, our analysis nests *Thaumastosaurus* within Frost et al.’s [18] Natatanura, for which the ossified stylus of the sternum may be a synapomorphy. More precisely, *Thaumastosaurus* is positioned as the sister taxon of a clade comprising the extant hyperossified *Ceratobatrachus*, *Aubria* and *Pyxicephalus*. This clade, recovered in all analyses of the four versions of the matrix (in addition to the analysis in which the first character, about the nasal, is scored from QU 17376), is supported by several synapomorphies: a medial contact of the nasals, cranial exostosis, articulation between the zygomatic ramus of squamosal and the maxilla, a contact between the maxillary arch and the anterior process of the vomer, and tall neural spines on anterior presacrals. However, many or all of these characters can arise convergently in hyperossified anurans and the placement of the Solomon Island *Ceratobatrachus* as the sister taxon of the African pyxicephalids, *Pyxicephalus* and *Aubria*, is not supported by any recent molecular analyses (e.g. [18], [41], [42], [43]). In the analysis that includes all characters, *Arariphrynus* from the Cretaceous (Aptian-Albian) of South America is also included within Ranoides and Natatanura, in contrast to its hyloid placement in Báez et al. [29]. In the cladogram, the presence of a contact between the pterygoid and the parasphenoid appears as a synapomorphy for both *Arariphrynus* and its sister group, but this contact occurs in a variety of other anurans, including some in our sampling. However, *Arariphrynus* is excluded from Ranoides in the trees yielded by our three other analyses, and the placement of this taxon should be treated with caution until further material is available.

In summary, the phylogenetic analysis places *Thaumastosaurus* within Ranoides [18] and, more specifically, within Natatanura [18]. As far as the diagnosis of Ranoides is concerned, two of Frost et al.’s characters cannot be checked in *Thaumastosaurus,* but the firmisternal condition of the pectoral girdle and possibly the ossification of the omosternum are regarded as synapomorphies of the clade [18]. The diagnosis of Natatanura provided by Frost et al. [18] is largely irrelevant here because the characters it includes are primarily larval features that cannot be documented in most extinct taxa. However, in addition to larval characters, Frost et al. [18] suggested that the ossified stylus of the sternum (metasternum for Frost et al.), a character present in *T*. *gezei*, may be a synapomorphy of Natatanura.

This is the first time that natatanuran ranoid affinities have been proposed for *Thaumastosaurus.* Ceratophryids [10], *Calyptocephalella* [12], or a South and Central American assemblage of various taxa [14] were previously suggested as the closest relatives of *Thaumastosaurus* based on skull characters alone. Two characters revealed in the mummy QU 17279 (pectoral girdle firmisternal and eighth vertebra amphicoelous rather than procoelous) argue against the latter referrals.

### Geological Age

Identification of the mummy QU 17279 as *T. gezei* sheds light on its geological age, which was uncertain as the specimen came from the ‘old collections’ of the Quercy Phosphorites [5]. As both QU 17279 and QU 17376 belong to the same species, they are probably of similar age, namely dating from the late Middle Eocene to the latest Eocene (MP 16–MP 19 or 20), the age proposed for QU 17376 [10], i.e. late Bartonian or Priabonian (about 40–34 Ma [44]).

### Palaeobiogeography

As noted above, most previous analyses placed *Thaumastosaurus* within Hyloides, closely related to South or Central American taxa [10], [12], [14]. This presented something of a biogeographical anomaly. However, the addition of new postcranial data has revealed that *Thaumastosaurus* is a natatanuran ranoid, a clade generally agreed to have African roots (e.g. [45]). Relationships between anurans from the Eocene of Europe (*Thaumastosaurus*) and African lineages would not be unexpected because evidence of interchanges between Europe and Africa during the Eocene has been reported for mammals and squamates [46].

## Conclusion

This study highlights the value of modern imaging technologies such as X-ray tomography to observe previously inaccessible anatomical features without destroying the original material. In this case, it has yielded important new information on a fossil frog specimen which, for 140 years, was known solely on the basis of its exceptionally well preserved external morphology. The mummified type of *Rana cadurcorum* Filhol, 1876 [7] is shown to be attributable to *Thaumastosaurus gezei* Rage and Roček, 2007 [10], previously known only from partial skull remains. The exquisite preservation of the mummy QU 17279 reveals new cranial and postcranial characters that permit a radical reassessment of the phylogenetic position of this problematic genus, showing it to be a ranoid with African affinities rather than a hyloid of South American origin. This, in turn, has solved a long-standing biogeographical enigma.

## Supporting Information

Data Matrix S1 Matrix used for the first analysis, in Mesquite NEXUS format. Matrix with all characters.
(NEX)

Data Matrix S2 Matrix used for the second analysis, in Mesquite NEXUS format. Same as supplementary on-line material S1 but with forelimb characters of *Thaumastosaurus* scored as unknown.
(NEX)

Data Matrix S3 Matrix used for the third analysis, in Mesquite NEXUS format. Same as data matrix S1, but without character 52 (‘sternum expansion of the coracoid’).
(NEX)

Data Matrix S4 Matrix used for the fourth analysis, in Mesquite NEXUS format. Same as data matrix S1, but with forelimb characters of *Thaumastosaurus* scored as unknown and without character 52 (‘sternum expansion of the coracoid’).
(NEX)

Character List S1 List of characters used in data matrix S1–S2 (S3 and S4 lack character 52).
(NEX)
