# Correction: A Re-Interpretation of the Eocene Anuran *Thaumastosaurus* Based on MicroCT Examination of a ‘Mummified’ Specimen

**DOI:** 10.1371/annotation/f7988d67-24b9-493c-9aef-c5c715948a1e

**Published:** 2013-12-20

**Authors:** Fabien Laloy, Jean-Claude Rage, Susan E. Evans, Renaud Boistel, Nicolas Lenoir, Michel Laurin

The file linked to "Character List S1" is incorrect. The correct file can be viewed here: http://www.plosone.org/corrections/pone.0074874.001.cn.doc

